# Suitability of issuing sickness certifications in remote consultations during the COVID-19 pandemic. A mixed method study of GPs’ experiences

**DOI:** 10.1080/02813432.2023.2282587

**Published:** 2024-02-07

**Authors:** Elin Breivik, Eli Kristiansen, Paolo Zanaboni, Monika A. Johansen, Nicolas Øyane, Trine Strand Bergmo

**Affiliations:** a Norwegian Centre for E-health Research, University Hospital of North Norway, Tromsø, Norway; b Centre for Quality Improvement in Medical Practices, Bergen, Norway; c Department of Global Public Health and Primary Care, University of Bergen, Norway; d Department of Pharmacy, UiT The Arctic University of Norway, Tromsø, Norway

**Keywords:** e-health, remote consultation, primary health care, sickness certification, qualitative research, quantitative research

## Abstract

**Objective:**

To explore Norwegian GPs’ experiences with and perceived suitability of issuing sickness certifications in remote consultations during the COVID-19 pandemic.

**Design:**

We used a mixed methods research design. An online survey with 301 respondents was combined with qualitative interviews with ten GPs.

**Setting:**

Norwegian general practice.

**Results:**

Most GPs agreed it was difficult to assess a patient’s ability to work without physical attendance for a first-time certification in remote consultations. However, extending a certification was considered less problematic. If physical examinations were required, the GPs would ask the patient to come to the office. The most suitable diagnoses for remote certification were respiratory infections and COVID-19-related diagnoses, as well as known chronic and long-term diseases. The GPs emphasized the importance of knowing both the patient and the medical problem. The GP-patient relationship could be affected by remote consultations, and there were mixed views on the impact. Many GPs found it easier to deny a request for a sickness certification in remote consultations. The GPs expressed concern about the societal costs and an increased number of certifications if remote consultations were too easily accessible. The study was conducted during the COVID-19 pandemic, and the findings should be interpreted in that context.

**Conclusions:**

Our study shows that issuing sickness certifications in remote consultations were viewed to be suitable for COVID-19 related problems, for patients the GP has met before, for the follow-up of known medical problems, and the extension of sickness certifications. Not meeting the patient face-to-face may affect the GP-patient relationship as well as make the GPs’ dual role more challenging.

## Abstract (plain language summary)

The GPs perceived issuing sickness certifications in remote consultations as suitable when patient and health problem are known, and when the certification is an extension.

Issuing sickness certifications in remote consultations can both harm and strengthen the GP-patient relationship.

The GPs were aware of their social responsibility and were concerned that issuing sickness certificates in remote consultations can change their sick-listing practice.

## Introduction

During the COVID-19 pandemic, a temporary regulation allowed general practitioners (GPs) in Norway to issue sickness certifications in remote consultations. The rationale was primarily to minimize the spread of COVID-19, to secure adequate patient care, and relieve the GPs’ increasing workload [1].

Sickness certification is a common task in general practice [2]. Previous studies have indicated that GPs experience tasks and situations related to sickness certification as problematic [3,4]. GPs are responsible for issuing sickness certifications based on clinical judgement of patients [5]. This includes assessing disease-related work disability, estimating work-disability duration and degree of absence, and assessing the advantages and disadvantages together with the patient [3,6]. Such tasks are complex and difficult for the GPs [3,7–9], especially when it is challenging to set an accurate diagnosis [10,11] or the patients’ complaints lack objective clinical evidence [4,10]. In these cases, many GPs feel obligated to trust their patients’ unverifiable symptoms [12]. Consequently, GPs are increasingly experiencing a role conflict between satisfying the interest of their patients and the doctor-patient relationship on one side [13] and on the other side acting as society’s gate keepers with the obligation to ration access to health care and welfare benefits [2]. This dual role can lead to conflicts with patients when the GPs must deny a demand of sickness certification [4,6,8,14].

All Norwegian inhabitants are entitled to be registered as a patient with a GP through the regular GP scheme, which is regulated by the municipalities. The scheme is governmentally funded, and patients pay a small fee [15]. The GPs are gatekeepers to specialities and welfare benefits [16]. Sickness certifications beyond self-certifications of three to eight days (depending on employer) must be certified by a GP either inside or outside the regular GP scheme or other health care personnel entitled to issue sickness certifications [17]. Overall, 85% of all sickness certificates are issued by GPs [1].

The GPs are recommended to offer remote consultations, but it is not mandatory. Remote consultations with the GP are defined as consultations that are conducted at distance, either synchronously (telephone consultations or video consultations) or asynchronously (text-based e-consultations). Remote consultations are delivered through the national health portal Helsenorge.no or through private digital platforms [18]. In recent years physicians outside the regular GP scheme (‘online doctors’) offer remote consultations that are payable in full by the patients.

GPs report confidence in decision making in remote consultations when it concerns known patients [19–21], existing conditions [20,22,23], monitoring [24,25], simple problems [20] or problems not requiring physical examination [25,26]. To safely issue a sickness certification in a remote consultation the GP must assess whether the information about the patient’s condition provided remotely is sufficient to assess the patient’s work disability. Little research has been conducted on the issue of sickness certifications in remote consultations. The temporary regulation introduced in Norway during the COVID-19 pandemic accentuated the need to investigate how sickness certifications can be issued in remote consultations to ensure appropriate assessment of the patient’s work disability and address the patient’s needs, while supporting at the same time the GP’s duty as gatekeeper [27]. This study addresses some of these issues.

The aim of this study was to explore Norwegian GPs’ experiences with and perceived suitability of issuing sickness certifications in remote consultations during the COVID-19 pandemic.

## Materials and methods

### Study design

We used a mixed methods research design to address the study aim. An online survey of GPs’ experiences was combined with qualitative interviews to explore GPs’ views on the suitability of issuing sickness certifications in remote consultations during the COVID-19 pandemic.

### Online survey

An online survey was conducted to gather GPs’ opinions on different claims about consequences and suitability of issuing sickness certifications in remote consultations. The survey was a mandatory part of a continuous education and quality improvements course for GPs focused on sickness certifications, organized by the Centre for Quality Improvement in Medical Practices (SKIL) and was answered by 301 course participants. The course including the survey took place between 26 August 2020 and 22 April 2021. The survey was developed by all authors and consisted of nine questions. Three questions central to the current study addressed the following issues: assessment of the patient’s ability to work in first-time sickness certifications (5-point Likert scale), the extension of sickness certifications without physical attendance (5-point Likert scale) and the ability to deny requests if conditions for assessing work ability were not met through remote consultations (5-point Likert scale). In addition, a non-mandatory free text question explored the GPs’ opinions about suitable diagnoses/problems for issuing sickness certifications in remote consultations. The respondents could provide up to five diagnoses/problems. Background variables for the respondents (gender, age, authorisation year, specialization, patient list length, vacancies, permanent or temporary appointment, and county) were available from the course database. However, some variables were not available for all participants, and they could not be linked to the respondents’ answers. Both survey and background characteristics were anonymous. Data describing the general GP population was extracted from the General Practitioner Statistics 2020 (updated 31 December 2020) available from the Directorate of Health and used to evaluate the representativeness of the survey respondents. The survey data were analysed by EK and PZ.

### In-depth interviews

We conducted in-depth interviews with GPs to explore their experiences and attitudes towards issuing sickness certifications in remote consultations in more detail. We developed a semi-structured interview guide with open-ended questions. The guide concentrated on criteria for appropriate clinical assessment of the patient, the GPs’ experiences of issuing sickness certifications in remote consultations and perceived impact on the GPs, the patients, and the society at large. The interview guide was developed by EB, EK, and TSB.

All course participants received an e-mail in November 2020 sent by the course organizer (SKIL) with an invitation to participate in an individual interview. Of the 26 GPs who responded and were willing to be interviewed, six GPs withdrew. We consecutively included GPs in a random manner. Data saturation was reached after ten interviews, when no new significant information was obtained [28].

The interviews were conducted at a distance from December 2020 to February 2021 due to the societal lockdown. As all informants had experience with remote communication and lived across the country, we considered this to be an appropriate solution. All interviews were conducted in video conference calls except one that was conducted by telephone due to problems with the internet connection. The GPs were offered reimbursement for the time spent in the interview. All interviews were conducted and recorded by EB and transcribed verbatim by a professional agency.

The analysis was performed by researchers with background in health science and health service research and experience with qualitative research methods. The transcribed texts were read and coded separately by two authors (EB and TSB) who subsequently participated collectively in the analyses of the data using systematic text condensation [29]. In this process, units of meaning concerning the suitability of issuing sickness certifications in remote consultations were identified and categorized into thematic subgroups. The content of the subgroups was reduced and summarized to condensates, that were described in continuous text to contain the essence of each subgroup. We grouped the subgroups into three recurring themes: clinical appropriateness, GP-patient relationship, and societal responsibility. The software programme Nvivo 12 was used to organise the data in the process of analysis. The quotes were translated from Norwegian to English by the authors.

### Ethical considerations

No personally identifiable information was recorded in either the survey or the interviews. All data were analysed anonymously. Participation in the survey was necessary for all the course participants to complete the course. Participation in the interviews was voluntary. Ethics approval from the Regional Committees for Medical and Health Research Ethics was deemed not necessary according to the Health Research Act. The study was approved by the Data Protection Officer of the University Hospital of North Norway.

## Results

### Online survey

307 GPs signed up to the course, while 301 completed and gave their opinion on the three claims about issuing sickness certifications in remote consultations (Table 1). It is not possible to identify who completed the course.

**Table 1.** Background characteristics of the respondents.

Background characteristics	*n*	%
Gender		
Female (%)	172	56.0%
Male (%)	134	43.6%
N/A	1	0.3%
Age		
< 30 years (%)	7	2.3%
30–39 years (%)	108	34.5%
40–54 years (%)	134	43.0%
55–66 years (%)	50	16.0%
>67 years (%)	2	0.7%
N/A	11	
Years of authorization		
1980–1989 (%)	25	8.1%
1990–1999 (%)	31	10.1%
2000–2009 (%)	90	29.3%
2010–2020 (%)	122	39.7%
N/A	39	12.7%
Specialization in general medicine (%)	190	61.9%
N/A	39	12.7%
Length of patient list (patients)	1054	
Average number of available places at list (patients)	29	
Working as a substitute GP (%)	27	8.8%
N/A		3.6%
County of GP practice		
Agder (%)	22	7.2%
Innlandet (%)	0	
Møre og Romsdal (%)	35	11.4%
Nordland (%)	0	
Oslo (%)	46	15.0%
Rogaland (%)	32	10.4%
Troms og Finnmark (%)	28	9.1%
Trøndelag (%)	23	7.5%
Vestfold og Telemark (%)	27	8.8%
Vestland (%)	28	9.12%
Viken (%)	65	21.2%
N/A	1	0.3%

 Over two-thirds (68.4%, 206/301) of the respondents agreed (totally or partly) that it was difficult to assess the patient’s ability to work when issuing a first-time sickness certification without physical attendance. However, extending a sickness certification was to a larger extent unproblematic. More than half (59.8%, 180/301) of the respondents agreed (partly or totally) that extending a sickness certification in a remote consultation was normally unproblematic. About two-thirds of the respondents (65.2%, 196/301) agreed (partly or totally) that it was easier to deny a request for a sickness certification when it was done without physical attendance if the request did not meet the conditions for a sickness certification (Table 2).

**Table 2.** GPs’ claims about the suitability of issuing sickness certification without physical attendance.

Claim	*n*	%
First-time sickness certification without physical attendance makes it difficult to assess the patient’s ability to work		
Totally agree	64	21.3%
Partly agree	142	47.2%
Neither agree nor disagree	45	15.0%
Partly disagree	37	12.3%
Totally disagree	13	4.3%
Extension of sickness certification without physical attendance is normally un-problematic		
Totally agree	37	12.3%
Partly agree	143	47.5%
Neither agree nor disagree	57	18.9%
Partly disagree	53	17.6%
Totally disagree	11	3.7%
If the patient requests a sickness certification without meeting the conditions for a sickness certification – how does the form of consultation affect the ability to deny the request?		
Much easier to deny without physical attendance	60	19.9%
A bit easier to deny without physical attendance	136	45.3%
No difference	48	15.9%
A bit easier to deny with physical attendance	22	7.3%
Much easier to deny with physical attendance	23	7.6%
I don’t know	12	4.0%

A total of 896 free-text answers were provided by 298 GPs to the question regarding which diagnoses/problems were suitable for issuing sickness certificationss in remote consultations. The answers were divided into two main categories: specific diagnoses (*n* = 609) and patient or situation-specific problems (*n* = 287) (Table 3). Respiratory infections, COVID-19 related problems and mild mental disorders were the most stated diagnoses for which issuing sickness certifications in remote consultations worked well. Other suitable issues described were musculoskeletal complaints and other infectious diseases. Cancer, pregnancy-related problems, and migraines were also mentioned, however to a smaller extent. Many of the respondents who did not provide any specific diagnoses referred to a known health problem as suitable. In addition, many of the other statements were indirectly related to a known problem (e.g. extension of a sickness certifications, sickness certifications during or after a hospital stay and/or rehabilitation stay, long-term sick leave for chronically ill patients).

**Table 3.** Diagnosis or issues perceived suitable by GPs for issuing sickness certification without physical attendance.

Diagnoses (*n* = 609)	Frequency
Respiratory infections	153
COVID-19 related diagnoses	108
Mild mental disorders and exhaustion	108
Musculoskeletal complaints and follow-up on fractures	77
Infectious disease	70
Cancer	40
Pregnancy-related issues	29
Migraine	24
Patient or situation-specific problems (*n* = 287)	
Known health problems	57
Extension of sickness certification	55
Sickness certification during or after stay in hospital or rehabilitation institution	54
Chronic sick patients and long-term sickness	40
Patients wating for hospital stay	22
Earlier clarified and defined sickness certification process	20
Known patient	14
Other issues	25

### In-depth interviews

All ten informants were Norwegian GPs. Four of the GPs were men, six were women. Their experience as a GP varied from three to 35 years. Three informants used text-based e-consultations and video consultations before the COVID-19 pandemic, while seven had no previous experience and started with remote consultations after lockdown 12 March 2020.

Three themes regarding the suitability of remote consultations to issue sickness certifications were identified: clinical appropriateness, GP-patient relationship, and societal responsibility.

### Clinical appropriateness

The GPs had essentially similar experiences and perceptions about necessary conditions to make a safe and appropriate clinical assessment when issuing sickness certifications in remote consultations. These conditions applied regardless of which forms of remote consultation (telephone, video or text) they preferred or used.

Most GPs reported they knew their patients well and argued that prior knowledge of the patient was a necessary condition. In this context, a known patient means that the GP has met the patient several times before. Unknown patients had to come to the clinic for a face-to-face consultation.

I think that what is most important is that you know the patient. Because if there is someone I do not know, know who they are or know what attitude they have regarding sick leave, then it is much more difficult than with patients I have known for 20 years (GP9).

The GPs reported that issuing sickness certifications remotely were best suited for follow-up of a known medical problem and an already established plan for treatment. These plans could also have been made by specialists, such as in the case of cancer treatment or fractures, or if the patient was in a rehabilitation program. The GPs emphasized that most sickness certifications issued in a remote consultation concerned an extension of a sick leave.

Most sickness certifications that I issue in remote consultations concern follow-up. Then we have made a plan and I ask for feedback on how this plan has worked (GP3).

Some GPs expressed that in long-lasting sick leaves remote consultations could be useful to follow up the patients more closely and frequently, and be beneficial to both the GPs and the patients. However, it was emphasized that sickness certifications should not be issued for a long period without seeing the patient in person at the office.

The GPs were to some degree concerned about missing symptoms or information about the patients’ condition in remote consultations. They needed to be able to rule out serious illness or exacerbation of a known medical problem. If there was a need for a physical examination, the patient was asked to come to the GP’s office for a face-to-face consultation. Moreover, if they were in doubt, the patient could receive a sickness certification for a short period and be told to come the office for a physical consultation within a few days.

Yes, that’s when you feel like you’re not getting the big picture. Difficult to assess. What I usually do then … is that they get a sickness certification, I also say ‘OK, then I schedule you for a physical examination in five days’ (GP10).

The GPs strongly emphasized that sickness certifications in remote consultations were useful to improve continuity of care during the pandemic and to avoid patients with symptoms of COVID-19.

Yes, I would say that these are the cases where it would be absolutely essential to issue sickness certifications without personal attendance. Now you are not allowed to go to work if you have a cold, cough, or sore throat (GP1).

### GP-patient relationship

Although the GPs emphasized that they generally trusted the patients’ own report of symptoms, there were situations when they questioned the accuracy of the information provided in remote consultations. In these cases, several of the GPs insisted on face-to-face follow-up consultation.

It’s obvious that it costs more to sit and lie to my face than to do it on the phone (GP7).

One GP pointed out that work ethics was important to understand if a patient abused the system regardless of whether the certification was issued in a remote consultation or not. Some GPs expressed that the patients’ previous history of sick leave was important because it gave information of their attitude towards returning to work. If the patient wanted to return to work, the GPs were more confident that issuing the sickness certification was correct.

I use my gut feeling, then. If I feel that this is a person who is motivated to return to work, then it is really easy to issue sickness certification (GP7).

The GPs reported that conflicts between GPs and patients regarding the need for sickness certification happened regardless of consultation mode. However, they felt that for some patients it could be easier to request sickness certifications in remote consultations when the GP wasn’t present in the same room. At the same time, while it is in general difficult for GPs to deny a patient’s request for sickness certification, several of the respondents felt that it was easier to do so in a remote consultation.

I think it might be a little easier to deny a sickness certification on video then, than in person at the office. Actually (GP6).

Several GPs preferred physical consultations when issuing sickness certifications. Having the patient in the office provided extra information and improved both dialogue and relationship between doctor and patient and at the same time made the patient feel better attended to.

You lose all the intertextuality that is present in a face-to-face consultation setting. There is nothing, in my opinion, that replaces having the patient in front of you in the office. Both in terms of what the GP might get out of it […], but also how the patient experiences the GP (GP4).

Other GPs reported that, in general, they communicated well with the patients in remote consultations, and that giving the patient information in connection with the sickness certification was uncomplicated.

### Societal responsibility

The GPs had mixed views about whether their sick-listing practice changed during the period they have been allowed to issue sickness certificates in remote consultations. Some believed that the total number of issued sickness certifications did not increase, other claimed the opposite. However, most GPs expressed worry that an easier and more convenient access to GP consultations through remote tools may lead to an increased number of issued sickness certifications, and thus increased societal costs.

I think that if it [issuing sickness certifications in remote consultations] is generally allowed, there will be more sickness certifications (GP9).

The GPs were concerned that if physicians outside the GP scheme could issue sickness certifications in online consultations, patients could consult them in cases when their own GP refused to issue a certification. The GPs didn’t want to manage extension of sickness certifications initially certified by another physician.

Knowing the patient is very important in this job. It is probably easier to issue sickness certification in remote consultation if you have no follow-up responsibility or anything (GP6).

## Discussion

The GPs perceived the issuing of sickness certifications in remote consultations to be useful and sensible during the COVID-19 pandemic, in particular when the patient and the health problem were known, and when the sickness certification was an extension of a previously issued certification. Known chronic and long-term conditions, respiratory diseases, and COVID-19-related problems were considered problems that were suitable. Most of the survey respondents found it easier to deny requests for sickness certifications in remote consultations than face-to-face when the GP assessed that the conditions for sickness certifications were not met. Many of our informants worried that the GP-patient relationship might deteriorate when the consultation is not carried out face-to-face. Moreover, the GPs were aware of their social responsibility and were concerned that issuing sickness certifications in remote consultations could cause issuing too many sickness certifications.

Our results on the appropriateness of issuing sickness certifications in remote consultations resonate with the findings from previous studies on the suitability of remote consultations in general. These found that an established patient-doctor relationship [19,20] and a previously known medical problem [22,23] were important to make safe decisions in remote consultations. Furthermore, our respondents also reported that remote consultations were most suitable for the extension of sickness certifications. More than two thirds of our survey respondents agreed that it was difficult to assess the patient’s ability to work in case of a new medical problem. In contrast, almost 60% found it unproblematic to extend a sickness certificate without physical attendance. This is in line with the findings of a recent Norwegian study on GPs’ experiences with video consultation [21,23]. The interviewed GPs confirmed that they would not issue a sickness certification without prior knowledge of the patient and the medical problem and thus found that remote consultations are particularly suitable for the follow-up of chronic and long-term diseases. However, many of our survey respondents reported that, in addition to chronic and long-term diseases, respiratory infections and COVID-19-related problems were suitable for sickness certification in remote consultations (51% and 36% of the respondents, respectively). This study was conducted during the COVID-19 pandemic when remote consultations were often used to deliver sickness certifications due to restrictions to visit the GP office, for instance for patients in quarantine

Remote consultations may represent a safety challenge because of the inability to perform clinical examinations and difficulties in assessing both physical signs and non-verbal signals to inform clinical decision-making [30]. This view is confirmed by many of our informants. However, in situations characterized by diagnostic uncertainty, we found that the GPs normally required patients to attend an office visit to perform physical examinations. They therefore expressed little fear of missing serious illness when issuing sickness certifications in remote consultations. This is in line with Greenhalgh et al. [31], who observed that there is no need to physically examine every patient, and the GPs bring the patients in for assessment when needed.

Issuing sickness certifications is a challenging task regardless of whether the GPs meet the patient face-to-face or in a remote consultation. Several studies show that trust and knowledge of the patient are key factors on which GPs base their decisions when issuing sickness certification [10,32]. GPs report that it is particularly challenging when there is a lack of physical findings [6,11] and when they have to trust their patients’ story [10]. According to our respondents, this does not change in remote consultations: the GP must trust the patient regardless of whether the consultation is carried out face-to-face or in a remote consultation. Furthermore, our informants pointed out that the decision to issue sickness certifications in remote consultations is supported by the GP’s prior knowledge of the patient and the patient’s work ethics. The GPs in our interview study stated to know their patients well.

The GPs were concerned with safeguarding the relationship with their patients but had mixed views on how it was affected by issuing sickness certifications in remote consultations. Many experienced that remote consultations facilitated the maintenance of the GP-patient relationship for patients with chronic diseases, as well as improved continuity of care during the COVID-19 pandemic. Other preferred face-to-face consultations and worried that patients felt less seen and attended to in remote consultations. The quality of the dialog could also be reduced as non-verbal information could be missed. This contradiction may be owing to both context (consultation mode, patient characteristics, and study setting) and GP preferences. Several studies confirm that remote consultations have the potential for maintaining and strengthening [19,23,30] as well as harming [19,25,33] the GP-patient relationship.

Several studies have reported that in addition to safeguarding alliance and trust in the relationship with their patients, GPs are concerned with disagreements and conflict with patients when discussing sickness certifications [10,14,34]. As in line with other studies [8,10], our informants addressed that it is often felt uncomfortable to deny patients’ request for sickness certificates in face-to-face consultations. Our study suggests that it seems to be less uncomfortable for many GPs to deny patients requests when the patients are not present face-to-face. Almost two-thirds of the survey respondents agreed that it is easier to say no to patients in remote consultations than in face-to-face consultations. This finding is confirmed by Gomez [25], who reported that physicians sometimes feel more comfortable refusing patient requests during telemedicine visits than face-to-face. Possible explanations are that remote consultations have weakened the GP-patient relationship and that patients’ dissatisfaction may not feel as strong or personal at a distance.

Issuing sickness certifications is an important part of medical practice and our informants were deliberate about the task of manoeuvring between individual wishes and societal demands to manage access to welfare benefits. This is confirmed in other studies [34–36]. There are concerns that if the GPs are permanently permitted to issue sickness certifications in remote consultations, this will lower the threshold for patients to request inappropriate sickness certifications. Moreover, most informants in our study claimed to not have changed their own sickness certification practice, but they acknowledged that it may be more difficult to assess the patients’ ability to work in remote consultations. Further research is needed to investigate whether the possibility to issue sickness certifications in remote consultations in normal situations will lead to an increased volume of sickness certifications or more sickness certifications of longer duration.

Our informants reported that issuing sickness certificationss in remote consultations was sensible and useful during the pandemic, but it could become too easy. This is in line with what previous research has pointed out, that even though remote consultations are useful in many situations, many GPs prefer face-to-face consultations [37]. Consequently, while most GPs were positive about a permanent permission to issue sickness certifications in remote consultations, it should be restricted to situations where both the patient and the problem are known to the GP. The temporarily regulation allowing the issuing of sickness certifications in remote consultations was made permanent 1 July 2023, with the conditions that the patient and the medical problem is prior known to the GP, the patient’s ability to work can be assessed with professional soundness without physical attendance. Exceptions can be made to avoid the spread of public hazardous infectious disease. This policy change is in concordance with findings in this paper.

### Limitations

The main limitation of this study is that the data were collected during the COVID-19 pandemic. This could have made our informants more positive to issuing sickness certifications in remote consultations. Nevertheless, the study points at conditions for how the issue of sickness certifications in remote consultations can be recommended in a pandemic-free situation. Further research carried out in a post-pandemic period will be able to provide more knowledge about the issue of sickness certifications in remote consultations.

The respondents of both the survey and the interviews were recruited within a course on sick leaves and may have been more interested in the challenges of sick-listing practice than the general GP population, with a consequent possible sampling bias. Compared to the national GP population [Supplement material Table 1], there was a higher proportion of female among the respondents of the survey. Moreover, the respondents were slightly younger and not evenly distributed throughout the country compared to the national distribution.

The GPs involved in this study used different modes of remote consultation (telephone, text-based and video consultations), and few had experience with remote consultation before lockdown occurred. We did not collect data and analyse the results by forms of remote consultation and could therefore not draw conclusions on the suitability of each consultation form for sickness certification.

## Conclusions

This study shows that issuing sickness certifications in remote consultations is suitable for patients the GP has met before, where the GP follow up known medical problems, for example chronic and long-term conditions, in addition to COVID-19-related problems. The majority of the respondents agreed that extending a sickness certification in remote consultations was considered unproblematic. However, the GP-patient relationship may be impaired, and many GPs found that it could be easier to deny a request for a sickness certification in a remote consultation. There were also concerns about changed sickness certification practice. Further research is needed to explore the long-term effects of remote consultations on sickness certification practices.

## Supplementary Material

Supplemental Material
