# Supplementary material for: Suitability of issuing sickness certifications in remote consultations during the COVID-19 pandemic. A mixed method study of GPs’ experiences
Source: Scand J Prim Health Care. 2024 Feb 7;42(1):7–15. doi: 10.1080/02813432.2023.2282587 (PMC10851799; doi:10.1080/02813432.2023.2282587)
Supplement: Supplemental Material [file IPRI_A_2282587_SM2880.docx]

Supplement table 1: National background variables for GPs 2020.

| **Background characteristics** | **National GP statistics 2020** |
| --- | --- |
| Gender |  |
| Female (%) | 45.8% |
| Male (%) | 54.2% |
|  |  |
| Age (average years) | 47.2 |
| < 30 years (%) | 1.8% |
| 30–39 years (%) | 27.6% |
| 40–54 years (%) | 42.0% |
| 55–66 years (%) | 23.6% |
| > 67 years (%) | 4.9% |
|  |  |
| Year of authorization |  |
| 1980-1989 (%) | n/a |
| 1990-1999 (%) | n/a |
| 2000-2009 (%) | n/a |
| 2010-2020 (%) | n/a |
|  |  |
| Specialization in general medicine (%) | 63.2% |
|  |  |
| Length of patient list (patients) | 1052 |
| Average number of available places at list (patients) | 41 |
| Working as a substitute GP (%) | 3.5% |
|  |  |
| County of GP practice |  |
| Agder (%) | 6.0% |
| Innlandet (%) | 7.2% |
| Møre og Romsdal (%) | 5.3% |
| Nordland (%) | 5.5% |
| Oslo (%) | 11.0% |
| Rogaland (%) | 8.5% |
| Troms og Finnmark (%) | 6.0% |
| Trøndelag (%) | 9.0% |
| Vestfold og Telemark (%) | 7.8% |
| Vestland (%) | 12.5% |
| Viken (%) | 21.3% |
